# Development of Effective Lipase-Hybrid Nanoflowers Enriched with Carbon and Magnetic Nanomaterials for Biocatalytic Transformations

**DOI:** 10.3390/nano9060808

**Published:** 2019-05-28

**Authors:** Renia Fotiadou, Michaela Patila, Mohamed Amen Hammami, Apostolos Enotiadis, Dimitrios Moschovas, Kyriaki Tsirka, Konstantinos Spyrou, Emmanuel P. Giannelis, Apostolos Avgeropoulos, Alkiviadis Paipetis, Dimitrios Gournis, Haralambos Stamatis

**Affiliations:** 1 Biotechnology Laboratory, Department of Biological Applications and Technologies, University of Ioannina, 45110 Ioannina, Greece; renia.fotiadou@gmail.com (R.F.); mpatila@cc.uoi.gr (M.P.); 2 Department of Materials Science and Engineering, Cornell University, Ithaca, NY 14853, USA; mah424@cornell.edu (M.A.H.); ae276@cornell.edu (A.E.); epg2@cornell.edu (E.P.G.); 3 Department of Materials Science and Engineering, University of Ioannina, 45110 Ioannina, Greece; dmoschov@cc.uoi.gr (D.M.); tsirka.kyriaki@gmail.com (K.T.); konstantinos.spyrou1@gmail.com (K.S.); aavger@uoi.gr (A.A.); paipetis@uoi.gr (A.P.); dgourni@uoi.gr (D.G.)

**Keywords:** hybrid nanoflowers, lipase, magnetic nanomaterials, biocatalysis, enzyme immobilization

## Abstract

In the present study, hybrid nanoflowers (HNFs) based on copper (II) or manganese (II) ions were prepared by a simple method and used as nanosupports for the development of effective nanobiocatalysts through the immobilization of lipase B from *Pseudozyma antarctica*. The hybrid nanobiocatalysts were characterized by various techniques including scanning electron microscopy (SEM), energy dispersion spectroscopy (EDS), X-ray diffraction (XRD), Raman spectroscopy, and Fourier transform infrared spectroscopy (FTIR). The effect of the addition of carbon-based nanomaterials, namely graphene oxide and carbon nanotubes, as well as magnetic nanoparticles such as maghemite, on the structure, catalytic activity, and operational stability of the hybrid nanobiocatalysts was also investigated. In all cases, the addition of nanomaterials during the preparation of HNFs increased the catalytic activity and the operational stability of the immobilized biocatalyst. Lipase-based magnetic nanoflowers were effectively applied for the synthesis of tyrosol esters in non-aqueous media, such as organic solvents, ionic liquids, and environmental friendly deep eutectic solvents. In such media, the immobilized lipase preserved almost 100% of its initial activity after eight successive catalytic cycles, indicating that these hybrid magnetic nanoflowers can be applied for the development of efficient nanobiocatalytic systems.

## 1. Introduction

Over the last decades, the immobilization of enzymes onto nanostructured supports has been extensively used and has facilitated their applications, owing to their easy handling and operational stability, as well as facile recovery and reusability of the biocatalysts, leading to more efficient bioprocesses [1,2]. Various nanostructured composite materials with extensive active surface areas and desirable pore sizes, such as nanoporous supports, nanofibers, nanoparticles, and carbon-based nanomaterials (e.g., nanotubes and graphene) have been proven to be effective in manipulating the nanoscale environment of biomolecules [3,4,5] and, as a consequence, their biological function and stability.

Organic-inorganic hybrid nanomaterials (nanoflowers) are a recently developed group of nanoparticles that schematically resemble plant flowers in a nanoscale range [6]. Hybrid nanoflowers (HNFs) have attracted a lot of interest over the last years as host platforms for immobilizing enzymes, owing to the higher surface-to-volume ratio compared to spherical nanoparticles, as well as to their simple, eco-friendly, and cost-effective synthesis [7,8]. Nanoflowers containing various enzymes have been usually prepared as enzyme-Cu_3_(PO_4_)_2_∙3H_2_O hybrids by combining copper sulfate (CuSO_4_) with enzymes in phosphate-buffer saline (PBS). Different HNFs mainly based on copper (II) and calcium (II) ions have been used to form complexes with enzymes and other proteins [9,10,11]. Moreover, the development of protein-embedded HNFs based on other metal ions, such as zinc (II), cobalt (II), and iron (II), was recently reported [12,13,14]. The formation of HNFs comprises the following steps: the nucleation and formation of primary crystals, the growth of these aggregates, and the complete formulation of nanoflowers [8]. During the first step, protein molecules form complexes with metal ions, primarily through the coordination between nitrogen atoms of the amide groups present in the protein backbone and the metal ion. These complexes provide sites for nucleation. The intramolecular interactions between the metal ion and the protein promote the anisotropic growth of nano-petals (step 2) and, consequently, the formation of a flower-like structure in which proteins serve as the glue that binds the petals together (step 3). The formation of these enzyme-embedded HNFs do not require harsh conditions and toxic reactants for their self-assembly; thus, the immobilization procedure is facilitated with biomolecules in a one-step process. Moreover, the incorporated enzyme is subjected to minor manipulation in comparison with other conventional immobilization procedures, thus retaining its biocatalytic activity [8].

The selection of the enzyme and the metal ions—as well as the pH, the temperature, and the incubation time—plays an essential role for the configuration and the catalytic efficiency of the enzyme-containing nanoflowers [8,15]. A variety of enzymes with biotechnological interest have been encapsulated in HNFs and successfully applied in dye decolorization [11,16], the production of esters [17], the detection of phenols or glucose [9,18], the degradation of pollutants [19], and the development of biosensors [20,21] in which two or more enzymes were successfully encapsulated in the same nanoflower.

The enhanced activity of enzymes that is observed in various HNFs is mainly attributed to their high surface area, which decreases mass-transfer limitations, along with the specific interactions of the enzyme molecules and metal ions [8,22,23]. However, the biocatalytic activity and stability of some HNFs are reduced by the interactions between metal ions and proteins [24]. Recently, it was proposed that the incorporation of surfactants [25], biopolymers such as chitosan [26], and carbon-based nanomaterials [27,28] could enhance the catalytic properties as well as the mechanical strength of enzyme-containing nanoflowers, leading to stable nanohybrids.

Herein, we describe the preparation of novel hybrid nanoflowers consisting of copper (II) or manganese (II) ions, combined with magnetic nanoparticles and carbon-based nanomaterials, and we investigate their use as versatile host platforms for the development of sufficient systems for the immobilization of enzymes. The addition of carbon-based nanomaterials, namely graphene oxide and multi-walled carbon nanotubes, in the preparation of nanoflowers is expected to provide high surface area and extraordinary mechanical properties, whereas the incorporation of magnetic nanoparticles, such as maghemite, allows the easy and quick separation of the nanoflowers by the application of an external magnetic force. The use of these novel HNFs as host platforms for the immobilization of lipase B from *Pseudozyma antarctica,* an enzyme with numerous biotechnological applications, was investigated. The novel nanobiocatalysts were characterized by scanning electron microscopy (SEM), energy dispersion spectroscopy (EDS), X-ray diffraction (XRD), Raman spectroscopy, and Fourier transform infrared spectroscopy (FTIR), while the effect of the composition of nanoflowers on the catalytic activity, thermal activity, and operational stability of the immobilized enzyme was investigated. Moreover, the ability of the lipase-based nanoflowers to catalyze the synthesis of lipophilic derivatives of phenolic antioxidants, such as tyrosol, in non-aqueous media, as well as in environmental-friendly ionic solvents, was also investigated.

## 2. Materials and Methods

### 2.1. Materials

Lipase B from *Pseudozyma antarctica* (formerly Candida antarctica, CaLB) was purchased from Novozymes A/S (Bagsværd, Denmark) and was utilized without further purification. 4-nitrophenyl butyrate (*p*-NPB), 4-nitrophenol (*p*-NP), copper (II) sulfate pentahydrate, manganese (II) sulfate, tyrosol, and dimethyl sulfoxide were obtained from Sigma–Aldrich (St. Louis, MO, USA). Vinyl butyrate was obtained from Fluka. The ionic liquid (IL) 1-Butyl-3-methylimidazolium hexafluorophosphate ([BMIM]PF_6_) with a purity of 97.0% was purchased from Sigma–Aldrich (St. Louis, MO, USA). Choline chloride (ChCl) and urea (U) were obtained from Sigma-Aldrich (St. Louis, MO, USA) and used for the preparation of deep eutectic solvents (DES), according to a previous work [17]. All organic solvents used were of analytical grade.

### 2.2. Preparation of CaLB Nanoflowers

The CaLB hybrid nanoflowers were prepared according to Ge et al. [8]. Typically, 0.42 mL of CuSO_4_ or MnSO_4_ aqueous solutions (120 mM) were added to 50 mL of phosphate buffer saline (PBS 1X, pH 7.4), which contained 0.4 mg mL^−1^ CaLB. The mixtures were placed for incubation at 25 °C for 3 days. The nanoflower precipitates were separated by centrifugation at 4000 rpm for 10 min, washed three times with distilled water, and dried under vacuum over silica gel at room temperature. Nanoflowers were stored at 4 °C until used. The prepared copper- and manganese-based samples are labeled Cu_3_(PO_4_)_2_ and Mn_3_(PO_4_)_2_, respectively.

For the preparation of nanomaterials-modified CaLB nanoflowers, a similar approach was followed. Graphene oxide (GO), oxidized multi-walled carbon nanotubes (CNTs), and maghemite nanoparticles (γ-Fe_2_O_3_) were synthesized as reported elsewhere [29,30,31]. Briefly, 5 mg of GO and 3 mg of oxidized CNTs or γ-Fe_2_O_3_ nanoparticles were added in 49 mL of PBS and sonicated for 20 min. After the dispersion of the nanomaterials, 1 mL of CaLB solution and 0.42 mL of CuSO_4_ or MnSO_4_ aqueous solutions (120 mM) were added into the mixture. The next steps were the same as those described previously. Nanoflowers containing only GO or CNTs were also prepared. The prepared modified copper-based samples are labeled GO-Cu_3_(PO_4_)_2_, CNTs-Cu_3_(PO_4_)_2_, GO/CNTs-Cu_3_(PO_4_)_2_, and GO/Fe_2_O_3_-Cu_3_(PO_4_)_2_, and the prepared modified manganese-based samples are labeled GO-Mn_3_(PO_4_)_2_, CNTs-Mn_3_(PO_4_)_2_, GO/CNTs-Mn_3_(PO_4_)_2_, and GO/Fe_2_O_3_-Mn_3_(PO_4_)_2_.

### 2.3. Characterization of CaLB Nanoflowers

SEM images were acquired from a JEOL JSM-5600 microscope (JEOL Ltd., Tokyo, Japan) with 10 and 25 kV accelerating voltage. Moreover, the surface morphologies of the samples were determined by field emission scanning electron microscopy (FE-SEM) using a SEM Zeiss Gemini 500 (Oberkochen, Germany). Prior to SEM analysis, the nanoflowers were placed in double-sided carbon tape and sputter-coated with gold-platinum. Phase elemental distribution was studied with SEM/EDS (JEOL JSM-6510 LV equipped with an X-Act EDS-detector by Oxford Instruments, Abingdon, Oxfordshire, UK).

The XRD patterns of all CaLB-HNFs were collected on a D8 Advance Bruker diffractometer with Cu Kα radiation (40 kV, 40 mA) and a secondary-beam.

Raman spectrocopy was used to confirm the presence of the carbon nanomaterials in the nanomaterials-modified CaLB-HNFs. The Raman spectra were recorded with the Labram HR system by HORIBA Scientific (HORIBA, Paris, France). The 514.5 green line of an air cooled Ar-Ion Laser was employed for the Raman excitation using a confocal aperture of 100. The laser power at the focal plane of the x100 objective was circa 0.8 mW. Spectral treatment included only a linear baseline subtraction.

FTIR was utilized to confirm the successful immobilization of CaLB in the nanoflower structure. The spectra were recorded in the range of 400–4000 cm^−1^ using a FTIR-8400 infrared spectrometer (Shimadzu, Tokyo, Japan) equipped with a deuterated triglycine sulfate (DTGS) detector. For each sample, a total of 64 scans were averaged, using a 2 cm^−1^ resolution. The samples were prepared using KBr pellets containing a circa 2 wt% sample. The similarity of FTIR spectra in the Amide I region (1600–1700 cm^−1^) was quantified by calculation of the correlation coefficient, r, using the following equation:

(1) $$r=\frac{\Sigma xiyi}{\sqrt{\Sigma xi^{2}\Sigma yi^{2}}},$$

where *x* and *y* represent the spectral absorbance values of the reference and sample spectra, respectively, at the *i*th frequency position [32]. For identical spectra, the *r* value is equal to 1.0, while spectra that have differences will show lower values.

### 2.4. Determination of Encapsulation Yield

The amount of the immobilized CaLB was determined by calculating the protein concentration present in the supernatant after the immobilization procedure using the Bradford assay [33]. Enzyme encapsulation was estimated as the difference between the initial amount of the enzyme and the amount of the enzyme in the supernatant after immobilization.

### 2.5. Activity of CaLB Nanoflowers

The activity of CaLB-HNFs was determined by the hydrolysis of *p*-NPB. Specifically, 0.5 mg of CaLB nanoflowers was added into 2 mL of phosphate buffer (50 mM, pH 7.5). The reaction was initiated with the addition of 20 μL of a 50 mM *p*-NPB solution (dissolved in DMSO), and the mixture was incubated for up to 10 min at 40 °C, 650 rpm. The 4-Nitrophenol (*p*-NP) release was monitored at 405 nm. The activity was estimated by measuring the concentration of *p*-NP using a standard curve. In this work, one unit of lipase activity was defined as the specific quantity of CaLB nanoflowers required to hydrolyze 1 μmol of *p*-NPB per reaction minute. Blank measurements without any enzyme were also incubated with the substrate for ten minutes, and their absorbance was measured where no catalytic activity was observed.

### 2.6. Stability of CaLB Nanoflowers

The thermal stability of free CaLB and CaLB-HNFs was tested at 60 °C for up to 24 h in phosphate buffer (50 mM, pH 7.5). In order to determine the remaining activity of CaLB nanoflowers, aliquots were taken at predetermined interval times for measuring the remaining lipase activity. The remaining hydrolyzing activity was estimated as described before, monitoring the increase in the absorbance of *p*-NP.

### 2.7. Transesterification of Tyrosol Catalayzed by CaLB Nanoflowers

The performance of CaLB-HNFs was tested on their ability to synthesize tyrosol esters. Typically, tyrosol (20 mM), vinyl butyrate (100 mM), and 4 mg mL^−1^ of CaLB-HNFs were added in various organic solvents and ionic liquids. The reaction mixtures were incubated for 72 h under stirring at 50 °C. Synthesis reactions were repeated twice, while experiments without nanoflowers were also conducted, and any decrease in the amount of tyrosol was observed for the selected solvents. The concentration of tyrosol in the reaction mixtures was quantified by high performance liquid chromatography (HPLC), equipped with a μBondapack C18 reverse phase column (particle size 10 μm, length 300 mm, diameter 3.9 mm) and a diode array UV detector. The elution was carried out with 40% water (containing 0.1% acetic acid) in methanol at a flow rate of 1 mL min^−1^ for 30 min. Tyrosol and its ester derivative were detected at 280 nm, while the column temperature was set at 35 °C. The conversion yield of the enzymatic transesterification was based on the decrease in the concentration of tyrosol, which was calculated using a tyrosol standard curve.

### 2.8. Reusability of CaLB Nanoflowers

The reusability of CaLB-HNFs was tested with respect to *p*-NPB hydrolysis for nine consecutive cycles. After each catalytic cycle, the samples were recovered by centrifugation at 1000 rpm for 2 min and excessively rinsed out three times with phosphate buffer (50 mM, pH 7.5). In the case of GO/Fe_2_O_3_-based hybrid nanoflowers, an external magnetic field was applied after each cycle and between washing procedures. The relative activity (%) was defined as the ratio of the remaining activity to the activity of the first cycle.

Magnetic hybrid nanoflowers (GO/Fe_2_O_3_-basedHNFs) were tested for their reusability on the transesterification of tyrosol in *tert*-butyl-methylether. Tyrosol (20 mM), vinyl butyrate (100 mM), and 4 mg mL^−1^ of GO/Fe_2_O_3_ CaLB-HNFs were added in 1 mL *tert*-butyl-methylether, and the reaction mixture was incubated for 72 h under stirring at 50 °C. The nanobiocatalytic system was separated from the reaction solution by an external magnetic field and washed twice with 1 mL of *tert*-butyl-methylether. The modified nanoflowers were applied to a new reaction solution and tested as described before for eight successive cycles.

## 3. Results and Discussion

### 3.1. Morphological and Structural Characterization of CaLB Nanoflowers

In the present work, HNFs based on copper (II) or manganese (II) ions were prepared by a simple method and used as nanosupports for the encapsulation of lipase B from *Pseudozyma antarctica* (CaLB). The effect of the enrichment of HNFs with graphene oxide sheets, oxidized multi-walled carbon nanotubes, and γ-Fe_2_O_3_ nanoparticles on the morphological, structural, and catalytic properties of HNFs was investigated.

**Figure 1:**
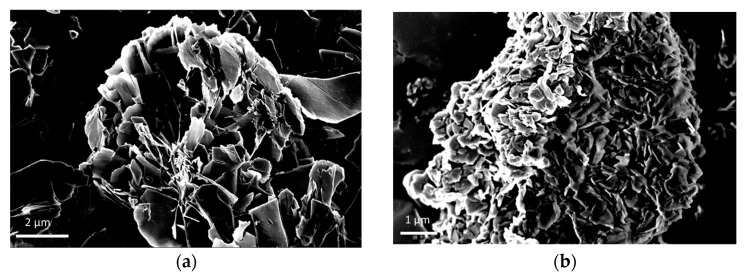

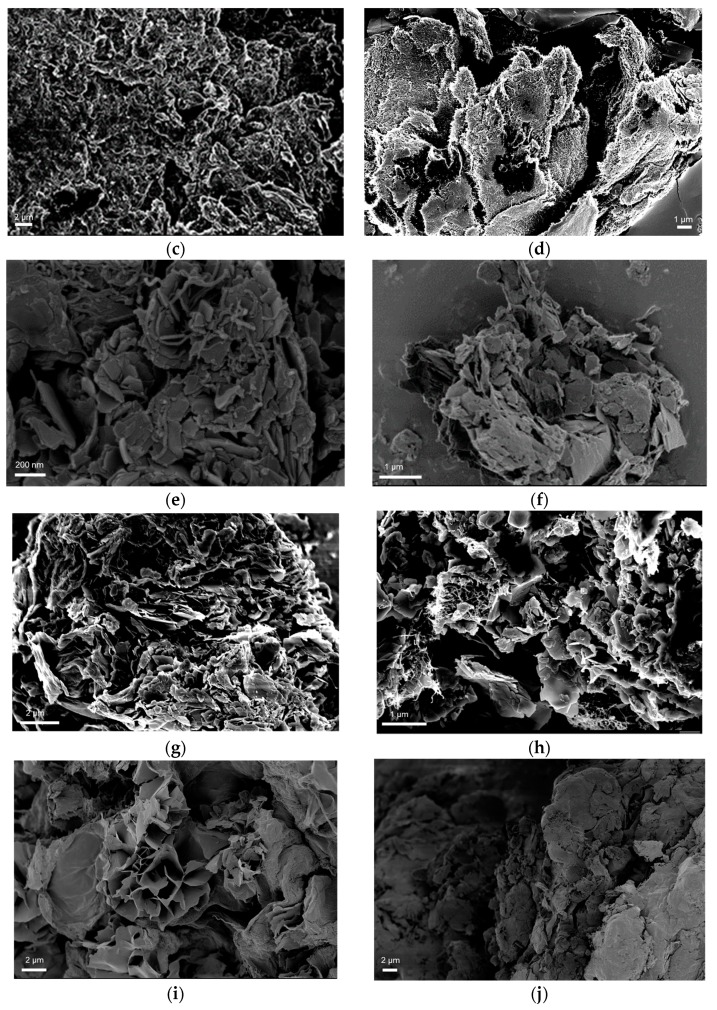
SEM images of: (**a**) unmodified Cu_3_(PO_4_)_2_ CaLB-HNFs; (**b**) unmodified Mn_3_(PO_4_)_2_ CaLB-HNFs; (**c**) GO-Cu_3_(PO_4_)_2_ CaLB-HNFs; (**d**) CNTs-Cu_3_(PO_4_)_2_ CaLB-HNFs; (**e**) GO/CNTs-Cu_3_(PO_4_)_2_ CaLB-HNFs; (**f**) GO/Fe_2_O_3_-Cu_3_(PO_4_)_2_ CaLB-HNFs; (**g**) GO-Mn_3_(PO_4_)_2_ CaLB-HNFs; (**h**) CNTs-Mn_3_(PO_4_)_2_ CaLB-HNFs; (**i**) GO/CNTs-Mn_3_(PO_4_)_2_ CaLB-HNFs; and (**j**) GO/Fe_2_O_3_-Mn_3_(PO_4_)_2_ CaLB-HNFs.

SEM images of unmodified Cu_3_(PO_4_)_2_ CaLB-HNFs revealed a high quality nanoflower formation with diameters in the range of 15–30 μm (Figure 1a). Moreover, SEM images of unmodified Mn_3_(PO_4_)_2_ CaLB-HNFs (Figure 1b) displayed a flower-like structure, though this structure was not as clear as in the case of Cu_3_(PO_4_)_2_. The nanomaterials-modified CaLB-HNFs, either with GO, CNTs, or both carbon structures together, exhibited different structures and formations, as indicated in Figure 1c–j, while a more detailed analysis can be found in the Supplementary material (Figure S1). It is noteworthy to add that the presence of carbon nanostructures (either GO or CNTs) in manganese-based nanoflowers facilitated the formation of nanoflowers in the final structures compared to the unmodified one (Figure 1g–j). Moreover, the combination of GO and CNTs resulted in the growth of clear crystals forming particular porous flower-like structures.

Modified GO/Fe_2_O_3_-based HNFs were further elementally analyzed using energy dispersion spectroscopy (EDS) (Figure S2). The peaks of carbon (C) and oxygen (O) were attributed to CaLB and the incorporated nanomaterials, while the presence of nitrogen (N) and sulfur (S) confirmed the successful encapsulation of the enzyme in the nanoflower structure. The appearance of copper (Cu) (Figure S2a), manganese (Mn) (Figure S2b) and phosphate (P) peaks indicated the successful formation of the nanoflowers. Sodium (Na) and chloride (Cl) peaks appeared due to the utilized preparation buffer. Moreover, the confirmation of the presence of iron nanoparticles (Fe) in the hybrid nanoflower structures was also observed—the atomic percentages of iron were 4.34 and 5.42% in the cases of GO/Fe_2_O_3_-Cu_3_(PO_4_)_2_ and GO/Fe_2_O_3_-Mn_3_(PO_4_)_2_ HNFs, respectively (Table S1).

X-ray diffraction (XRD) was used to characterize the unmodified and the nanomaterials-modified CaLB-HNFs, and the XRD patterns are provided in the Supplementary material (Figure S3). For the copper-based CaLB-HNFs, the XRD patterns represented peaks for the Cu_3_(PO_4_)_2_⋅3H_2_O (JCPDS 00-022-0548) phase, while for the manganese-based CaLB-HNFs, the phase of manganese changed from Mn_3_(PO_4_)_2_ for the unmodified nanoflowers to Mn_2_P_2_O_7_ for the nanomaterials-modified HNFs [34].

**Figure 2:**
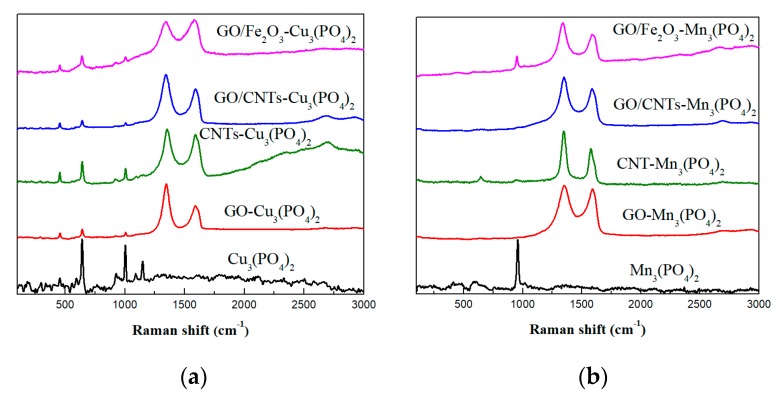
Raman spectra of: (**a**) Cu_3_(PO_4_)_2_-based CaLB-HNFs and (**b**) Mn_3_(PO_4_)_2_-based CaLB-HNFs.

The presence of the carbon-based nanostructures in the CaLB-HNFs was confirmed with Raman spectroscopy. The Raman spectra of the unmodified Cu_3_(PO_4_)_2_ CaLB-HNFs and Mn_3_(PO_4_)_2_ CaLB-HNFs, as well as the modified HNFs with GO, CNTs, and γ-Fe_2_O_3,_ are presented in Figure 2. The spectrum of the unmodified Cu_3_(PO_4_)_2_ CaLB-HNFs presented several vibrational modes; the most pronounced were located at 645 cm^−1^, 927 cm^−1^, and 1147 cm^−1^ and can be attributed to the antisymmetric bending of the PO_4_^3−^ ion, the symmetric stretching vibrations of PO_4_^3−^ ion, and the antisymmetric stretching vibrations of the PO_4_^3−^ ion, respectively [35]. The unmodified Mn_3_(PO4)_2_ CaLB-HNFs presented a strong vibrational mode at 958 cm^−1^ that can be ascribed to the symmetric stretching mode of the PO_4_^3−^ ion [36].

The preparation of Cu_3_(PO_4_)_2_- and Mn_3_(PO_4_)_2_-based CaLB-HNFs with GO in both led to the appearance of the characteristic of carbon-based materials vibrational modes, D and G, located at circa 1346 cm^−1^ and 1590 cm^−1^, respectively [37], while a weak asymmetric 2D vibrational mode was also present in both cases at around 2685 cm^−1^ [38]. Similarly, the preparation of both ion-based CaLB-HNFs with CNTs led to the appearance of the characteristic D and G vibrational modes located at circa 1350 cm^−1^ and 1587 cm^−1^, respectively, while the 2D vibrational mode of the CNTs was located at circa 2700 cm^−1^. The more intense D and G vibrational modes were also present when both GO and CNTs were added in the CaLB-HNFs, establishing the successful incorporation of the carbonaceous nanomaterials into the HNFs.

**Figure 3:**
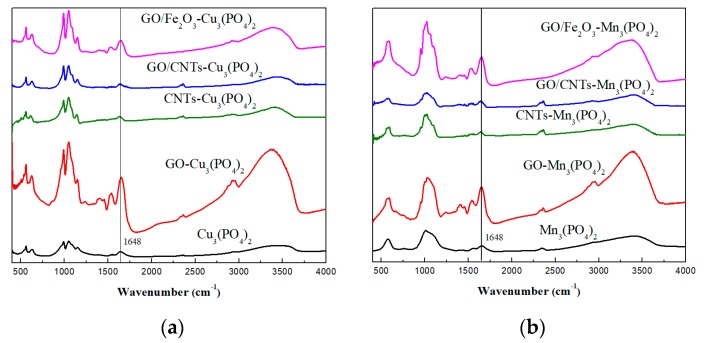
FTIR spectra of: (**a**) Cu_3_(PO_4_)_2_-based CaLB-HNFs and (**b**) Mn_3_(PO_4_)_2_-based CaLB-HNFs.

To confirm the successful immobilization of CaLB in the 3D nanostructures, all nanobiocatalytic systems were characterized by FTIR spectroscopy by recording the spectra in the range 400 cm^−1^ to 4000 cm^−1^. As seen in Figure 3, peaks at the region 950 to 1060 cm^−1^ were associated with the asymmetric stretching vibrations of PO_4_^3−^, while peaks at the region 550 cm^−1^ to 650 cm^−1^ arose from the bending vibrations of bridging phosphate groups, such as O-P-O [39,40]. The presence of CaLB in the nanoflower structure was confirmed by the peak at 1648 cm^−1^, which arises from the stretching vibrations of C = O of the peptide chain of the enzyme and corresponds to the Amide I band [41,42].

To better assess the dissimilarities among the spectra of the CaLB-HNFs, we compared the correlation coefficients (*r*) in the Amide I region (1600–1700 cm^−1^), according to previously published work [43,44,45]. As seen from Table 1, for most of the CaLB-HNFs, *r* was close to 1.0, indicating that CaLB was able to maintain its native secondary structure upon encapsulation in the nanoflower structure. GO has formed a cage-like structure in which lipase was encapsulated, preserving the secondary structure of the enzyme [27]. In contrast, when CaLB-HNFs were prepared with CNTs, especially when combined with GO, the changes in the *r* value were more pronounced. This result could be attributed to conformational changes occurred during the encapsulation of CaLB in the nanoflower structure. The disorder of the natural conformation of CaLB, may arise from the over-crowded enzyme molecules within the strongly packed GO/CNTs HNF structure.

### 3.2. Biocatalytic Characterization of CaLB Nanoflowers

The encapsulation yield and specific hydrolytic activity of all CaLB-HNFs are presented in Table 2. The protein loading for unmodified Cu_3_(PO_4_)_2_ and Mn_3_(PO_4_)_2_ CaLB-HNFs were 57.6% and 49.0%, respectively, while their specific activity was calculated at 8.3 and 96.7 U g^−1^, respectively, pointing out that the kind of the metal ion significantly affects the hydrolyzing ability of the immobilized lipase. It has been previously proposed that enzymes provide different binding sites for metal ions, and, as such, nucleation sites are formed in different enzyme regions, affecting the 3D structure and activity of the immobilized biocatalysts [15]. Moreover, in the case of Cu_3_(PO_4_)_2_ CaLB-HNFs, lipase could have been embedded deep inside the flower-like structure, preventing the active sites of the CaLB from interacting with the substrate and thus leading to low catalytic activity, due to steric hindrance phenomena [28,46].

In order to provide more binding sites for the formation of CaLB-HNFs, different carbon-based and magnetic nanomaterials were added to the hybrid nanostructures during the preparation procedure. All HNFs enriched with carbon or magnetic nanomaterials exhibited higher encapsulation yields than those without nanomaterials, regardless of the metal ion type. The highest encapsulation yields were observed when GO was used as an additive. For instance, the encapsulation efficiency reached up to 70.5 and 67.1% in the case of GO-Cu_3_(PO_4_)_2_ and GO-Mn_3_(PO_4_)_2_ CaLB-NHFs, respectively. Similar results have also been reported by Li and co-workers when GO was added in the formation of laccase-based nanoflowers [27]. CNTs also seem to affect the immobilization efficiency of CaLB, as is consistent with previous work [28]. The large surface area of GO and CNTs seems to increase the available binding sites and thus promote enzyme adsorption, in addition to stabilizing the 3D structure of the nanoflower. Moreover, the presence of oxygen-containing groups in the surface of these nanomaterials may result in the formation of electrostatic interactions between those functional groups and the copper cations, stabilizing the nucleation step.

The modification of CaLB-HNFs with carbon or magnetic nanomaterials enhanced the specific hydrolytic activity of the immobilized enzyme. In the case of manganese-based nanoflowers, the specific activity of the enzyme was increased up to around two-times in the presence of nanomaterials. The beneficial effect of the use of nanomaterials was more pronounced in the case of copper-based nanoflowers. More specifically, all the nanomaterials significantly outperformed in terms of activity the unmodified Cu_3_(PO_4_)_2_ nanoflowers. GO sheets, CNTs, and γ-Fe_2_O_3_ nanoparticles, due to randomly distributed oxygen-containing groups on their surface, interact with positively charged metals and amino groups on the enzyme’s surface, leading to more stable and active flower-like structures [28]. Such interactions could lead to a more active conformation [47,48]. Compared to each individual nanomaterial, the GO/CNTs hybrid system was not as beneficial as expected, maybe due to the uniform dispersion of lipase within the nanoflower structure or stereochemical hindrance. Moreover, CaLB immobilized on GO/CNTs nanoflowers presented the highest conformational changes (as previously discussed, Table 1), which could result in lower catalytic activity. It is important to mention that the preparation of HNFs containing both carbon nanomaterials and magnetic nanoparticles has not been previously reported. GO/Fe_2_O_3_-based HNFs reached high encapsulation yields, while GO/Fe_2_O_3_-Cu_3_(PO_4_)_2_ CaLB-HNFs exhibited one of the highest catalytic activities among all nanoflowers.

**Figure 4:**
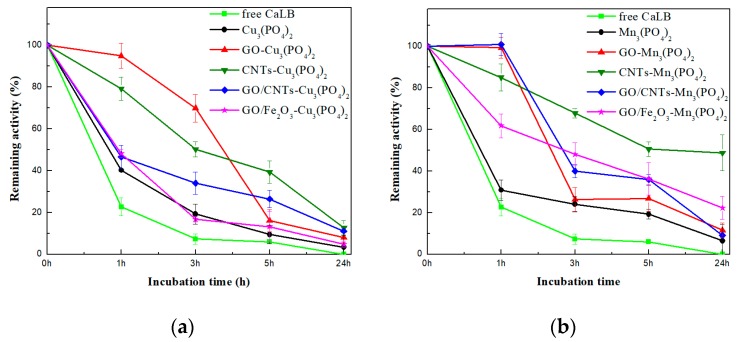
Thermal stability of: (**a**) Cu_3_(PO_4_)_2_-based CaLB-HNFs and (**b**) Mn_3_(PO_4_)_2_-based CaLB-HNFs at 60 °C. The 100% percentage corresponds to the activity at t = 0 min.

The thermal stability of the CaLB-HNFs was also investigated. The remaining hydrolytic activity was estimated after incubation of nanoflowers for up to 24 h in phosphate buffer at 60 °C, and is presented in Figure 4.

As seen in Figure 4, the use of hybrid nanoflowers as supports for the immobilization of CaLB increased the thermal stability of the immobilized enzyme. Specifically, the catalytic activity of free CaLB decreased to 20% after the first hour of incubation, while unmodified CaLB-Cu_3_(PO4)_2_ and Mn_3_(PO_4_)_2_ CaLB-HNFs retained up to 40% and 31% of their initial activity, respectively. Moreover, after 5 h of incubation, free CaLB was totally inactivated, while the immobilized lipase on unmodified HNFs retained up to 19.2% of their activity, indicating that the nanoflower 3D structure can protect the active conformation of the enzyme, thus enhancing its stability [49]. Similar observations have also been reported for lipase from the porcine pancreas [50]. The thermal stability of the immobilized CaLB was further improved when HNFs containing carbon and γ-Fe_2_O_3_ nanomaterials were used as immobilization supports. This observation could be attributed to the protective effect these nanomaterials offer on the stability of protein molecules [51,52]. Amongst the nanomaterials, CNTs stabilized the immobilized enzyme the most for both copper- and manganese-based nanoflowers (12.7% and 49% of enzyme activity, respectively, was retained after 24 h of incubation). Their high surface area, as well as the fact that CNTs are distributed within the petals of the flower-like structure, enables lipase to maintain its stability [27,28]. The conformational changes previously described (Table 1) may lead to a more rigid folding of lipase and thus enhance its stability [51]. Furthermore, in comparing the two inorganic components, it is clear that manganese HNFs exhibited higher remaining activity than copper HNFs, underlining the correlation of the different interactions developed between nanomaterials and each metal ion.

**Figure 5:**
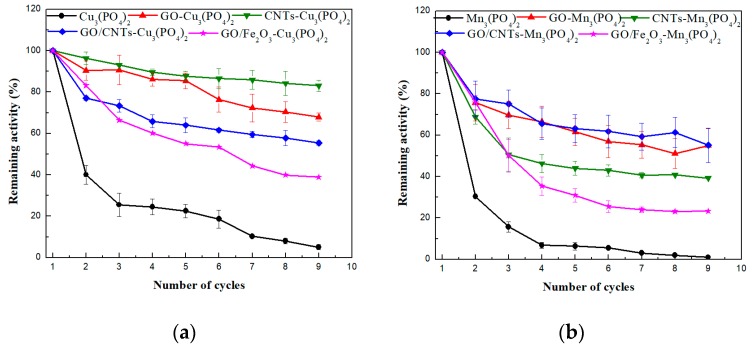
Operational stability of: (**a**) Cu_3_(PO_4_)_2_-based CaLB-HNFs and (**b**) Mn_3_(PO_4_)_2_-based CaLB-HNFs. The 100% percentage corresponds to the lipase hydrolytic activity at the first catalytic cycle.

One of the major drawbacks of using soluble enzymes in large-scale reactions is reusability, due to their incapability of maintaining their stability under harsh conditions, and the difficulty of removal from the reaction system, due to their high solubility. Therefore, the immobilization of enzymes enhances their stability and enables their separation and use in successive cycles, making them an asset for industrial applications. In the present study, the operational stability of the CaLB-HNFs was investigated for the hydrolysis of *p*-NPB, and the results are presented in Figure 5. As seen, unmodified Cu_3_(PO_4_)_2_ and Mn_3_(PO_4_)_2_ CaLB-HNFs were almost deactivated after the fifth biocatalytic cycle. It is possible that the disruption of the non-covalent bonds between the organic and inorganic parts of the nanoflowers in the aqueous environment accelerated the enzyme leaching or gradual degradation of the flower-like morphology, leading to low enzymatic activity, which is in agreement with that recently reported [40].

In the case of nanomaterials-modified HNFs, the operational stability of CaLB was notably increased. Immobilized CaLB on nanomaterials-based HNFs could be efficiently used for nine consecutive cycles for the hydrolysis of *p*-NPB. The residual activity of nanomaterials-modified Cu_3_(PO4)_2_ and Mn_3_(PO4)_2_ CaLB-HNFs retained up to 83% even after nine catalytic cycles. These results infer that the presence of nanomaterials in the nanoflowers protects the enzyme configuration, thus enhancing its stability for successive hydrolysis cycles. Similar to the thermal stability studies presented above, CNTs-modified CaLB-HNFs offered the most beneficial impact on the operational stability of CaLB, indicating that the incorporation of CNTs inside the nanoflower structure enables the adoption of a more rigid conformation of CaLB, stabilizing it against repeatable usage [53]. It is interesting to note that, although manganese-based HNFs presented higher thermal stability than copper-based HNFs (as previously discussed), their operational stability was lower compared to copper-based HNFs. It is possible that the enzyme leaching from the manganese-based HNFs is higher compared to copper-based HNFs, resulting in a higher loss of the residual activity of the enzyme.

### 3.3. Transesterification of Tyrosol by CaLB Nanoflowers in Non-Aqueous Media

The prepared nanomaterials-modified CaLB-HNFs were used for the synthesis of tyrosol esters in non-aqueous media. Tyrosol is a natural phenolic antioxidant derived from various plants, such as olive and green tea. This abundant product has been associated with many health-related benefits as well as plenty of industrial applications [54,55]. An increase of tyrosol lipophilicity is suggested to improve its antioxidant activity [56,57]. Thus, the enzymatic lipophilization of tyrosol may be of great interest. For this reason, GO/Fe_2_O_3_-Cu_3_(PO_4_)_2_ and GO/Fe_2_O_3_-Mn_3_(PO_4_)_2_ CaLB-HNFs were used as biocatalysts for the transesterification of tyrosol with vinyl butyrate (Figure S4) in a variety of organic solvents, as well as in environmentally friendly ionic and deep eutectic solvents; the results are presented in Table 3. GO/CNTs-based HNFs were also used for the transesterification of tyrosol; the results are presented in Table S2.

As seen in Table 3, both CaLB-HNFs were able to catalyze the transesterification of tyrosol, achieving high conversion yields in most of the non-aqueous solvents. It has been recently proposed that the hydrophobic surface of the hybrid nanoflowers benefits synthetic reactions in non-aqueous solvents by promoting the oriented delivery of the substrates near the hydrophobic surface of nanoflowers and, thus, to the active sites of the enzyme [49]. Conversion yields of transesterification seem to strongly depend on the nature of the organic solvent, namely its polarity and viscosity. More specifically, the nanoflower-catalyzed reactions in non-polar solvents, e.g., n-hexane and *tert*-butyl-methylether, exhibited high conversion yields up to 100%. Moreover, the reaction rate of the transesterification reaction catalyzed by GO/Fe_2_O_3_–Mn_3_(PO_4_)_2_ CaLB-HNFs in hexane and *tert*-butyl-methylether was up to 73-fold higher in comparison with that in other media (Table S3). Solvents with low polarity enable enzymes to preserve the essential water molecules bound on their surface in order to maintain their natural conformation and be fully functional [58,59]. On the other hand, the use of more hydrophilic solvents with higher affinity to interact with water molecules [60], such as 2-methyl-2-butanol and *tert*-butanol, led to a decrease of the conversion yield of the transesterification reaction.

Both HNFs were able to catalyze the transesterification of tyrosol in eco-friendly alternatives of organic solvents, such as ionic liquids and deep eutectic solvents ([BMIM][BF_6_] and ChCl:U, respectively). Those green solvents have been widely employed for enzymatic biotransformations, as they present high chemical and thermal stability, low vapour pressure, low toxicity, and the ability to enhance the catalytic performance of the enzymes [17,55,61]. As seen in Table 3, the conversion yield is decreased in the ionic liquid compared to organic solvents, which could be attributed to the low dispersability of the nanoflowers in these media. Moreover, the high viscosity of [BMIM][BF_6_] (381 cP at 25 °C)[62] and ChCl:U (1200 mPa s at 25 °C)[63] could lead to mass-transfer limitations, restricting the biocatalytic activity of immobilized CaLB [64,65].

**Figure 6:**
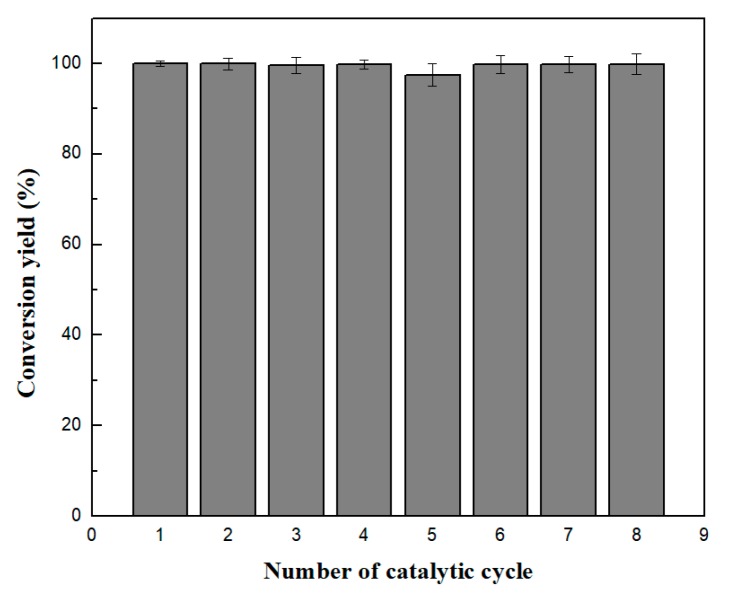
Operational stability of GO/Fe_2_O_3_-Mn_3_(PO_4_)_2_ CaLB-HNFs after eight reaction cycles for the enzymatic transesterification of tyrosol with vinyl butyrate in *tert*-butyl-methylether. Each reaction was carried out for 72 h at 50 °C.

The use of magnetic nanobiocatalysts could facilitate the separation from the reaction solution through the application of an external magnetic field and, thus, the reuse of the biocatalyst [52]. Considering this aspect, magnetic CaLB-HNFs were applied in consecutive reaction cycles for tyrosol transesterification. Figure 6 presents the remaining catalytic activity of the GO/Fe_2_O_3_-Mn_3_(PO_4_)_2_ CaLB-HNFs for successive catalytic cycles. As seen, these magnetic nanoflowers presented excellent operational stability after eight consecutive reaction cycles (576 h of total operation) without any loss of biocatalytic activity, making this hybrid nanobiocatalyst one of the most robust nanobiocatalysts reported until now for similar reaction processes. This enhanced operational stability, in comparison with the one described above for the hydrolysis of *p*-NPB (Figure 5), could be attributed to the fact that non-polar organic solvents do not remove protein-bound water that is crucial for maintaining protein structure and function, leading to a more rigid conformation of the immobilized biocatalyst [66,67].

## 4. Conclusions

Herein, we report the preparation and characterization of novel hybrid nanoflowers comprised of copper (II) or manganese (II) ions combined with magnetic nanoparticles and carbon-based nanomaterials. These nanoflowers can be effectively used as versatile host platforms for the immobilization of an industrially relevant enzyme (CaLB) through biomimetic mineralization. The metal ion and the nature of the nanomaterials affect the structural and catalytic characteristics of the immobilized lipase in different manners. The nanomaterials-modified hybrid nanoflowers presented an excellent catalytic performance in the production of tyrosol esters in different organic solvents and environmental-friendly ionic solvents. Furthermore, CaLB-magnetic HNFs (combining GO and maghemite nanoparticles) exhibited remarkable operational stability for the tyrosol transesterification reaction, as the nanobiocatalyst retained almost its entire catalytic activity even after eight successive reaction cycles, indicating that these bio-nanoconjugates could potentially be used as efficient tools for heterogeneous biocatalytic transformations in large-scale applications.

## Figures and Tables

**Table 1:** Correlation coefficient (*r*) between the FTIR spectra of CaLB-HNFs.

Nanoflower	*r*	Nanoflower	*r*
Cu_3_(PO_4_)_2_	0.976	Mn_3_(PO_4_)_2_	0.982
GO-Cu_3_(PO_4_)_2_	0.991	GO-Mn_3_(PO_4_)_2_	0.998
CNTs-Cu_3_(PO_4_)_2_	0.899	CNTs-Mn_3_(PO_4_)_2_	0.837
GO/CNTs-Cu_3_(PO_4_)_2_	0.879	GO/CNTs-Mn_3_(PO_4_)_2_	0.801
GO/Fe*_2_*O_3_-Cu_3_(PO_4_)_2_	0.991	GO/Fe*_2_*O_3_-Mn_3_(PO_4_)_2_	0.998

**Table 2:** Encapsulation yield and specific hydrolytic activity of various CaLB-HNFs.

Nanoflower	Encapsulation Yield (%)	Specific Activity (U g^−1^ Immobilized CaLB)
Cu_3_(PO_4_)_2_	57.6 ± 3.1	13.1 ± 0.5
GO-Cu_3_(PO_4_)_2_	70.5 ± 1.7	174.4 ± 0.7
CNTs-Cu_3_(PO_4_)_2_	57.5 ± 2.1	189.0 ± 3.9
GO/CNTs-Cu_3_(PO_4_)_2_	61.6 ± 1.5	167.0 ± 1.7
GO/Fe*_2_*O_3_-Cu_3_(PO_4_)_2_	59.0 ± 2.4	197.1 ± 2.5
Mn_3_(PO_4_)_2_	49.0 ± 1.7	161.2 ± 2.6
GO-Mn_3_(PO_4_)_2_	67.1 ± 3.6	284.7 ± 5.2
CNTs-Mn_3_(PO_4_)_2_	57.6 ± 1.2	175.6 ± 4.0
GO/CNTs-Mn_3_(PO_4_)_2_	65.9 ± 2.5	168.7 ± 1.0
GO/Fe*_2_*O_3_-Mn_3_(PO_4_)_2_	60.9 ± 2.7	175.9 ± 1.9

**Table 3:** Conversion yields for the enzymatic transesterification of tyrosol with vinyl butyrate in non-aqueous media catalyzed by GO/Fe_2_O_3_-Cu_3_(PO_4_)_2_ and GO/Fe_2_O_3_-Mn_3_(PO_4_)_2_ CaLB HNFs.

Reaction Medium	Conversion Yield (%)	
	GO/Fe_2_O_3_-Cu_3_(PO_4_)_2_ CaLB-HNFs	GO/Fe_2_O_3_-Mn_3_(PO_4_)_2_ CaLB-HNFs
n-Hexane	99.6 ± 0.4	100.0 ± 0.3
Acetonitrile	80.3 ± 0.3	80.7 ± 0.8
2-Methyl-2-butanol	30.2 ± 1.1	52.6 ± 1.2
*tert*-Butyl-methylether	98.9 ± 0.5	99.7 ± 0.6
*tert*-Butanol	23.2 ± 0.2	22.5 ± 0.4
[BMIM][PF_6_]	13.6 ± 1.6	20.0 ± 4.7
ChCl:U	33.2 ± 2.8	26.7 ± 4.6

## Supplementary Materials

The following are available online at https://www.mdpi.com/2079-4991/9/6/808/s1, Figure S1: SEM images of: (A1, A2) Cu_3_(PO_4_)_2_ CaLB-HNFs; (B1-B3) GO-Cu_3_(PO_4_)_2_ CaLB-HNFs; (C1-C3) CNTs-Cu_3_(PO_4_)_2_ CaLB-HNFs; (D1-D3) GO/CNTs-Cu_3_(PO_4_)_2_ CaLB-HNFs; (E1, E2) GO/Fe_2_O_3_-Cu_3_(PO_4_)_2_ CaLB-HNFs; (F1-F3) Mn_3_(PO_4_)_2_ CaLB-HNFs; (G1-G3) GO-Mn_3_(PO_4_)_2_ CaLB-HNFs; (H1,H2) CNTs-Mn_3_(PO_4_)_2_ CaLB-HNFs; (I1-I3) GO/CNTs-Mn_3_(PO_4_)_2_ CaLB-HNFs; (J1, J2) GO/Fe_2_O_3_-Mn_3_(PO_4_)_2_ CaLB-HNFs, Figure S2: EDS spectra of: (a) GO/Fe_2_O_3_-Cu_3_(PO_4_)_2_ CaLB-HNFs; (b) GO/Fe_2_O_3_-Mn_3_(PO_4_)_2_ CaLB-HNFs, Table S1: Elemental analysis of GO/Fe_2_O_3_-based CaLB-HNFs by EDS, Figure S3: XRD patterns of: (a) Cu_3_(PO_4_)_2_-based CaLB-HNFs; (b) Mn_3_(PO_4_)_2_-CaLB-HNFs, Figure S4: Transesterification of tyrosol with vinyl butyrate catalyzed by CaLB, Table S2: Conversion yields for the enzymatic transesterification of tyrosol with vinyl butyrate in non aqueous media, by GO/CNTs-Cu_3_(PO_4_)_2_ CaLB-HNFs, Table S3: Reaction rates (mM h^−1^) of tyrosol transesterification catalyzed by GO/Fe_2_O_3_-Mn_3_(PO_4_)_2_ CaLB-HNFs in non-aqueous media.
