# Supplementary material for: Development of Effective Lipase-Hybrid Nanoflowers Enriched with Carbon and Magnetic Nanomaterials for Biocatalytic Transformations
Source: Nanomaterials (Basel). 2019 May 28;9(6):808. doi: 10.3390/nano9060808 (PMC6632025; doi:10.3390/nano9060808)
Supplement: Supplementary file 1 [file nanomaterials-09-00808-s001.pdf]

nanomaterials

Supplementary Material

Development of effective lipase-hybrid nanoflowers enriched with carbon and magnetic nanomaterials for biocatalytic transformations.

Renia Fotiadou [1], Michaela Patila [1], Mohamed Amen Hammami [2], Apostolos Enotiadis [2], Dimitrios Moschovas [3], Kyriaki Tsirka [3], Konstantinos Spyrou [3], Emmanuel P. Giannelis[2], Apostolos Avgeropoulos[2], Alkiviadis Paipetis [3], Dimitrios Gournis and Haralambos Stamatis [1,*]

[1] Biotechnology Laboratory, Department of Biological Applications and Technologies, University of Ioannina, 45110 Ioannina, Greece; renia.fotiadou@gmail.com (RF), mpatila@cc.uoi.gr (MP)
[2] Department of Materials Science and Engineering, Cornell University, 14850 Ithaca, USA; mah424@cornell.edu (MAH), ae276@cornell.edu (AE), epg2@cornell.edu (EPG)
[3] Department of Materials Science and Engineering, University of Ioannina, 45110 Ioannina, Greece; dmoschov@cc.uoi.gr (DM), tsirka.kyriaki@gmail.com (KT), konstantinos.spyrou1@gmail.com (KS), aavger@uoi.gr (AA), paipetis@uoi.gr (AP), dgourni@uoi.gr (DG)

* Correspondence: hstamati@uoi.gr; Tel.: +30-265-100-7116

Received: 20 April 2019; Accepted: 23 May 2019; Published: date

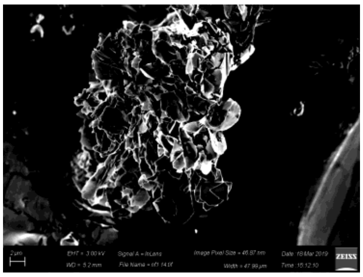

A1

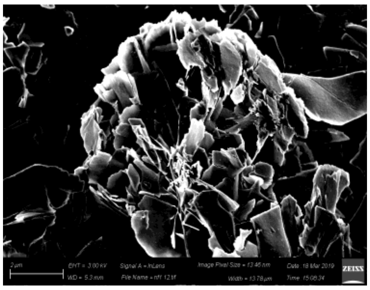

A2

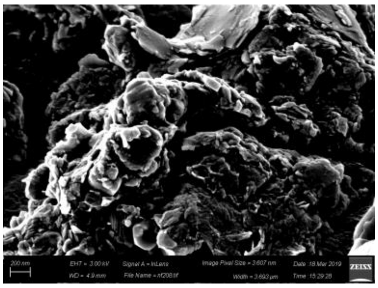

B1

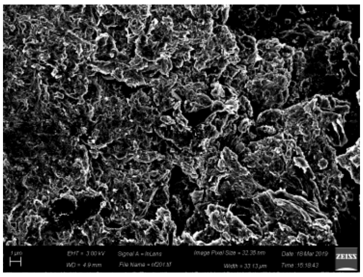

B2

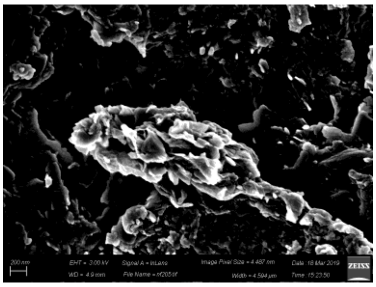

B3

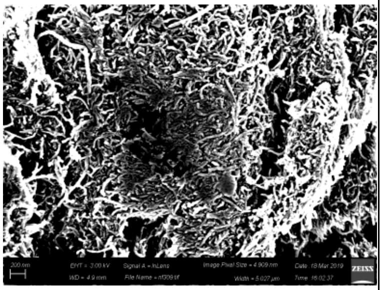 C1	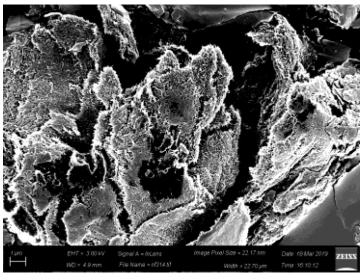 C2	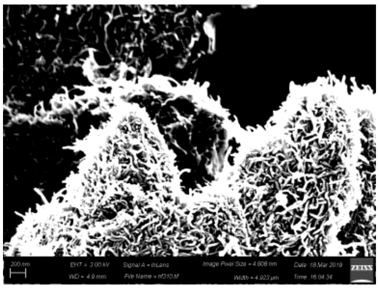 C3
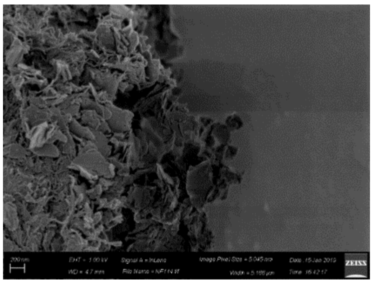 D1	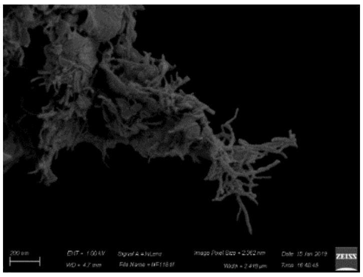 D2	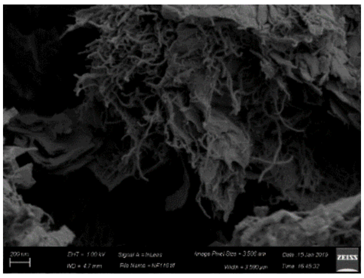 D3
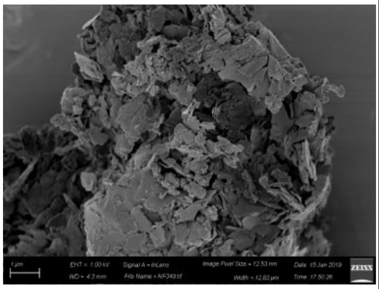 E1	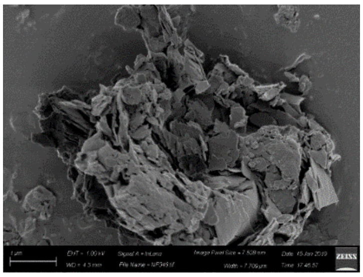 E2	
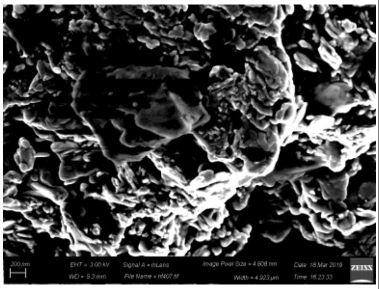 F1	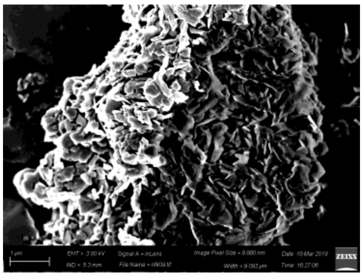 F2	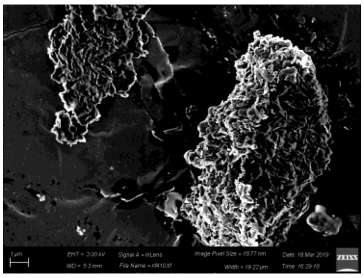 F3
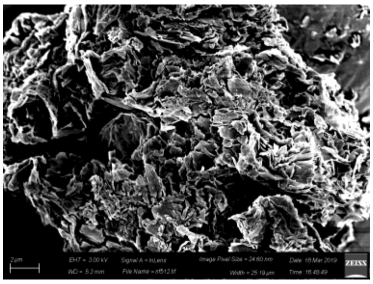 G1	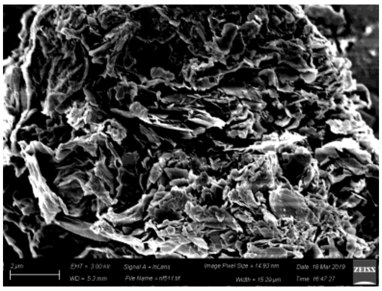 G2	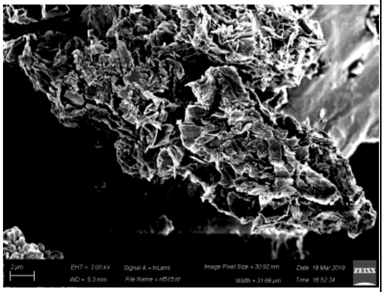 G3

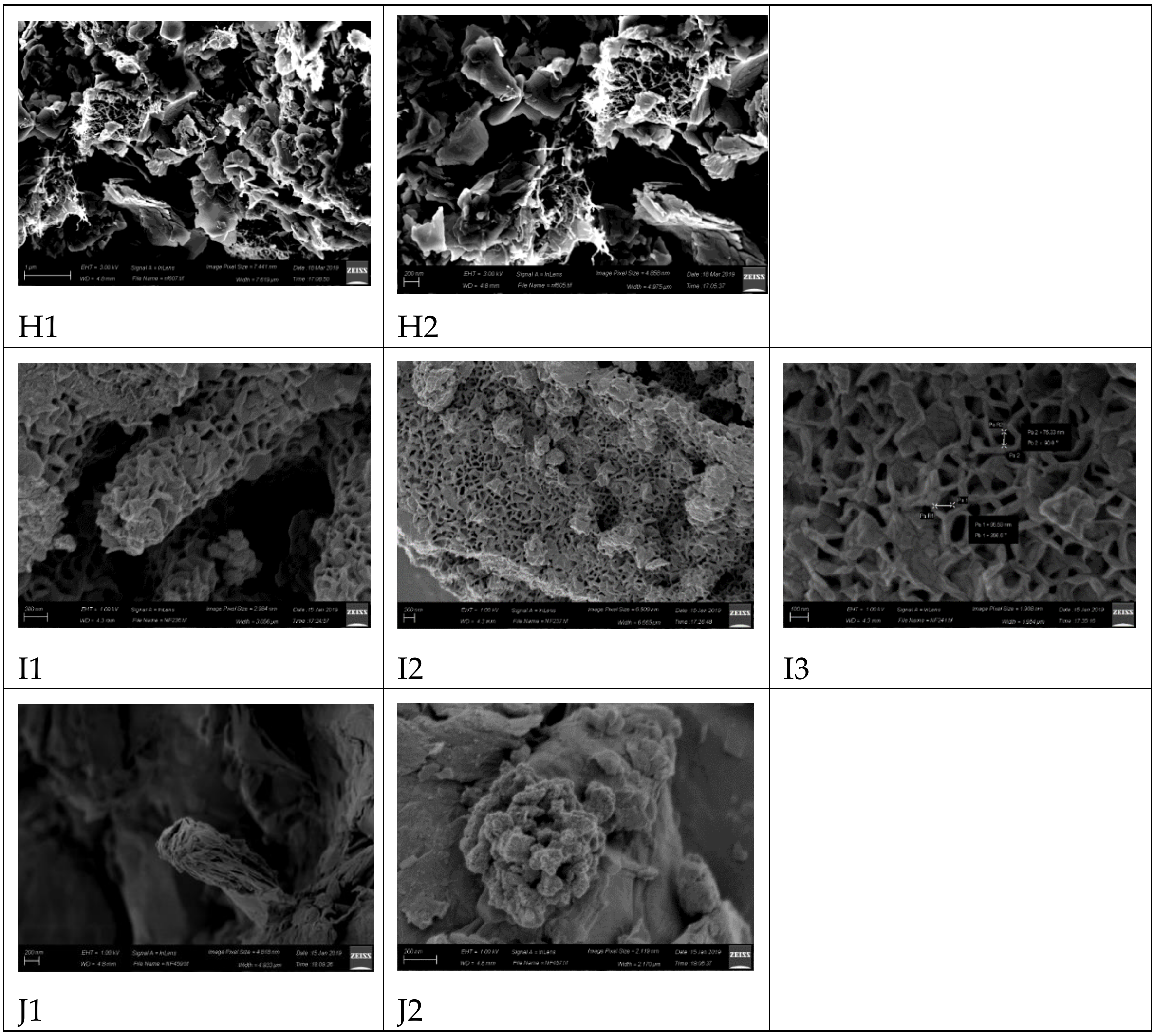

Figure S1. SEM images of: **(A1, A2)** $Cu_3(PO_4)_2$ CaLB-HNFs; **(B1-B3)** GO-$Cu_3(PO_4)_2$ CaLB-HNFs; **(C1-C3)** CNTs-$Cu_3(PO_4)_2$ CaLB-HNFs; **(D1-D3)** GO/CNTs-$Cu_3(PO_4)_2$ CaLB-HNFs; **(E1, E2)** GO/Fe_2O_3-$Cu_3(PO_4)_2$ CaLB-HNFs; **(F1-F3)** $Mn_3(PO_4)_2$ CaLB-HNFs; **(G1-G3)** GO-$Mn_3(PO_4)_2$ CaLB-HNFs; **(H1,H2)** CNTs-$Mn_3(PO_4)_2$ CaLB-HNFs; **(I1-I3)** GO/CNTs-$Mn_3(PO_4)_2$ CaLB-HNFs; **(J1, J2)** GO/Fe_2O_3-$Mn_3(PO_4)_2$ CaLB-HNFs.

Figure S1 presents the scanning electron microscope (SEM) images of CaLB-HNFs based on copper (II) (A1, A2) and manganese (II) (F1, F2, F3) ions. Regarding copper-based HNFs, Figures A1 and A2 reveal the presence of high quality of nanoflower structures with diameters in the range of 15–30 μm. After the addition of GO (B1-B3), the formation of a more aggregated structure is observed with flaky crystals, even in the case of the presence of magnetic nanoparticles (E1, E2). The presence of CNTs on the surface of the nanoflower structure provides further protection on the final flower nanostructures of CNTs-$Cu_3(PO_4)_2$ CaLB-HNFs (C1-C3). However, the presence of both GO and CNTs in the flower structures does not present a well-defined flower structure (D1-D3). On the other hand, in the case of manganese-based HNFs, the presence of carbon nanostructures, either GO (G1-G3) or CNTs (H1, H2), facilitates the formation of nanoflowers compared to the unmodified one (F1-F3). Notably, the combination of both nanostructures results in the growth of clear crystals forming particular flower porous structures (I1-I3 figures). Finally, the modified GO/Fe_2O_3-$Mn_3(PO_4)_2$ HNFs seem to adopt a more granular formation (J1, J2).

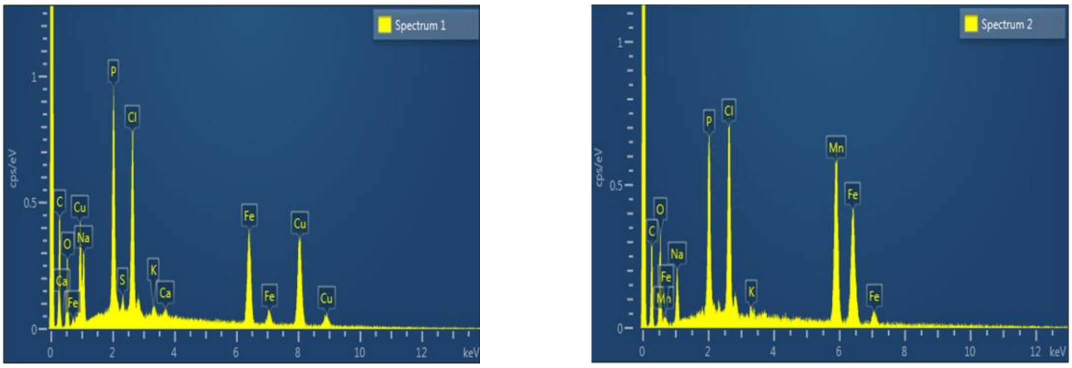

(**a**) (**b**)

Figure S2. EDS spectra of: (**a**) GO/Fe_2O_3-$Cu_3(PO_4)_2$ CaLB-HNFs; (**b**) GO/Fe_2O_3-$Mn_3(PO_4)_2$ CaLB-HNFs.

Table S1. Elemental analysis of GO/Fe_2O_3-based CaLB-HNFs by EDS.

**GO/Fe_2O_3-$Cu_3(PO_4)_2$ HNFs**	**Atomic %**	**GO/Fe_2O_3-$Mn_3(PO_4)_2$ HNFs**	**Atomic %**
C	59.01	C	57.99
O	15.38	O	17.39
Na	4.03	Na	3.07
P	5.16	P	4.05
S	0.39	S	0.0
Cl	3.98	Cl	4.20
K	0.22	K	0.19
Ca	0.18	Ca	0.0
Fe	4.34	Fe	5.42
Cu	7.31	Mn	7.69
Total	100.00	Total	100.00

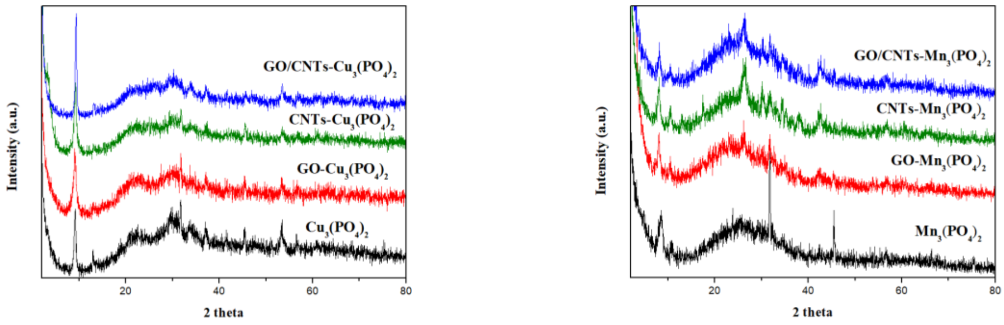

(**a**) (**b**)

Figure S3. X-ray diffraction patterns of: (**a**) $Cu_3(PO_4)_2$-based CaLB-HNFs; (**b**) $Mn_3(PO_4)_2$-based CaLB-HNFs.

XRD has been applied to all of the CaLB-HNF samples which were synthesized with copper or manganese ions and all patterns are shown in Figure S3. For the copper-based nanoflowers, the phase of $Cu_3(PO_4)_2{\cdot}3H_2O$ (JCPDS 00-022-0548) was detected, which was not changed when GO, CNTs or mixture of them were used for the preparation of the modified CaLB-HNFs (Figure S3a). Accordingly, in the case of manganese-based nanoflowers, a shift on the XRD peaks was observed, due to a phase change from $Mn_3(PO_4)_2$ to $Mn_2P_2O_7$ when the carbon nanostructures were added in the preparation of CaLB-HNFs (Figure S3b).

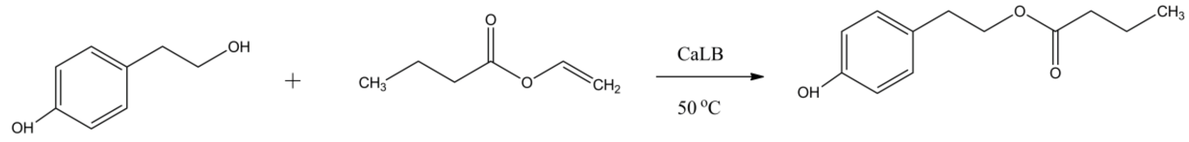

Tyrosol Vinyl butyrate Tyrosol ester

Figure S4. Transesterification of tyrosol with vinyl butyrate catalyzed by CaLB.

Table S2. Conversion yields for the enzymatic transesterification of tyrosol with vinyl butyrate in non-aqueous media, by GO/CNTs-$Cu_3(PO_4)_2$ CaLB-HNFs.

**Reaction medium**	**Conversion yield (%)**
n-Hexane	99.3 ± 0.2
Acetonitrile	82.4 ± 0.5
2-Methyl-2-butanol	65.8 ± 1.3
tert-Butyl-methylether	95.8 ± 0.3
tert-Butanol	35.2 ± 0.3
[BMIM][PF_6]	49.5 ± 3.6
ChCl:U	91.3 ± 3.8

Table S3. Reaction rates (mM h^{-1}) of tyrosol transesterification catalyzed by GO/Fe-$Mn_3(PO_4)_2$ CaLB-HNFs in non-aqueous media.

Reaction medium	Rate (mM h^{-1})
Hexane	9.50 ± 0.2
Acetonitrile	0.32 ± 0.8
2-Methyl-2-butanol	0.23 ± 0.7
tert-Butyl-methylether	8.25 ± 0.4
tert-Butanol	0.15 ± 0.4
[BMIM][PF_6]	0.13 ± 1.2
Chcl:U	0.14 ± 1.0

© 2018 by the authors. Submitted for possible open access publication under the terms and conditions of the Creative Commons Attribution (CC BY) license (http://creativecommons.org/licenses/by/4.0/).
